# A rare case of primary bladder malignant melanoma: a case report

**DOI:** 10.3389/fonc.2026.1785510

**Published:** 2026-05-19

**Authors:** Ying Yu, Xiangqian Nie

**Affiliations:** Zhejiang Provincial People's Hospital Bijie Hospital (The First People's Hospital of Bijie), Bijie, Guizhou, China

**Keywords:** bladder tumor, case report, melanoma, primary bladder melanoma, treatment

## Abstract

Primary melanoma of the bladder is an extremely rare malignant tumor. In recent years, only a few sporadic cases have been reported. We report a 59-year-old male patient who presented with gross hematuria and had bladder irritation symptoms such as frequent urination, urgency, and pain. CT imaging showed multiple exophytic small nodules on both sides of the bladder; under cystoscopy, multiple coral-like neoplasms were visible on both sides of the bladder, with no obvious bleeding on the surface, and the largest one was approximately 1.5 cm × 1.2 cm. No evidence of primary melanoma in other parts was found during the comprehensive examination. After the patient underwent transurethral resection of bladder tumor, he received regular intravesical instillation of epirubicin hydrochloride treatment. No recurrence was observed during the 5-month follow-up period. There were no more than 50 cases of primary bladder melanoma reported globally. Further immunohistochemistry should be performed as far as we know, this case is the first report of primary bladder malignant melanoma in Bijie City, Guizhou Province. The prognosis of this disease is generally poor, and its clinical diagnosis is relatively difficult. Immunohistochemistry plays a crucial role in its diagnosis. Due to insufficient understanding of it, there is currently no unified treatment standard.

## Introduction

Malignant melanoma (MM) is a type of tumor that originates from melanocytes in the neuroectoderm and has high invasiveness and metastatic potential. It mostly occurs in the skin (accounting for approximately 95%-96% of the primary site), but can also affect other epithelial tissues such as the gastrointestinal tract, eyes, and vagina (1–3).

Among the pathological types of bladder cancer, the most common one is urothelial carcinoma; primary melanoma of the bladder is extremely rare and is a highly invasive malignant tumor. Its clinical manifestations lack specificity, and its diagnosis mainly relies on immunohistochemistry and cystoscopy. The prognosis of patient is generally poor (4).

It was reported that this type of tumor accounted for less than 0.5% of all melanomas, and the total number of reported cases worldwide was less than 50 (3, 5). According to the published literature, there were currently no established treatment standards for localized or advanced primary melanoma.

The surgical treatment methods reported in the existing literature mainly include transurethral bladder tumor resection (TUR), partial bladder resection (PC), and radical bladder resection (RC) (6). However, recurrence and metastasis were likely to occur after the surgery, and the overall prognosis remained poor. The early diagnosis of this disease was difficult, and patients often presented with gross hematuria and lower urinary tract symptoms as the initial manifestations. We reported that a case of primary melanoma of the bladder and described its clinical features, immunohistochemistry, and molecular analysis results, aiming to provide a reference for a better understanding of the disease and appropriated treatment.

## Case presentation

**Figure 1:**
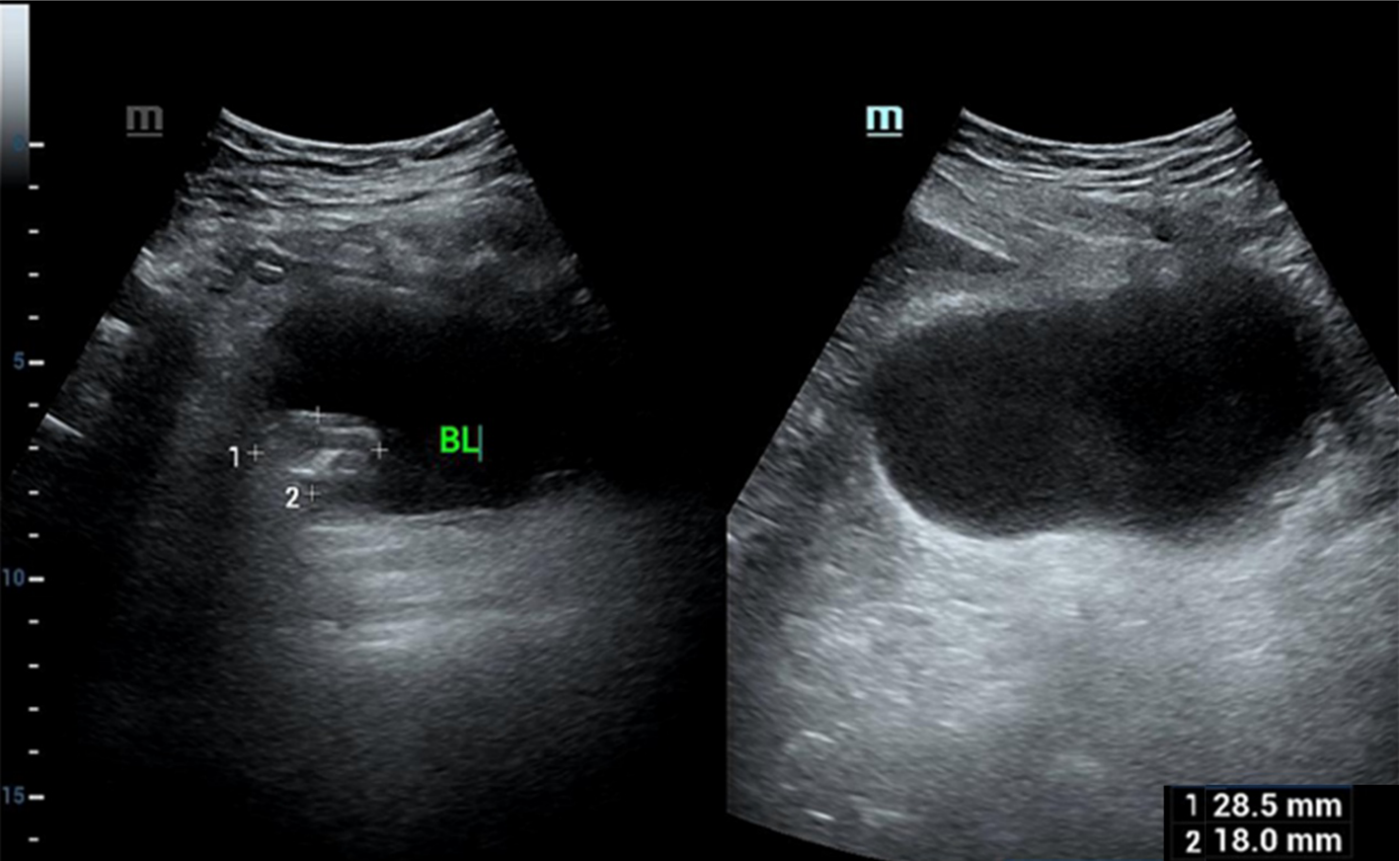
The size of the mass in the bladder is 2.85 × 1.8 cm.

**Figure 2:**
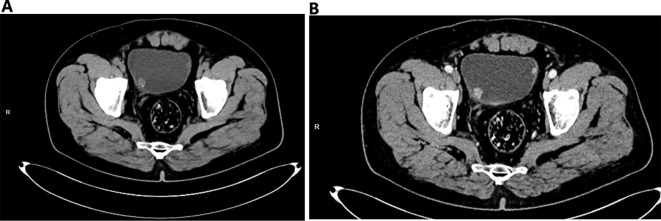
**(A)** Several nodular and slightly hyperdense shadows can be seen on both sides of the bladder (one on the right and two on the left). The lesions have narrow bases and are connected to the bladder wall. Spot calcification shadows can be observed at the edges. **(B)** During the arterial phase and venous phase of the enhanced scan, gradual enhancement occurs, and the enhancement decreases in the delayed phase.

**Figure 3:**
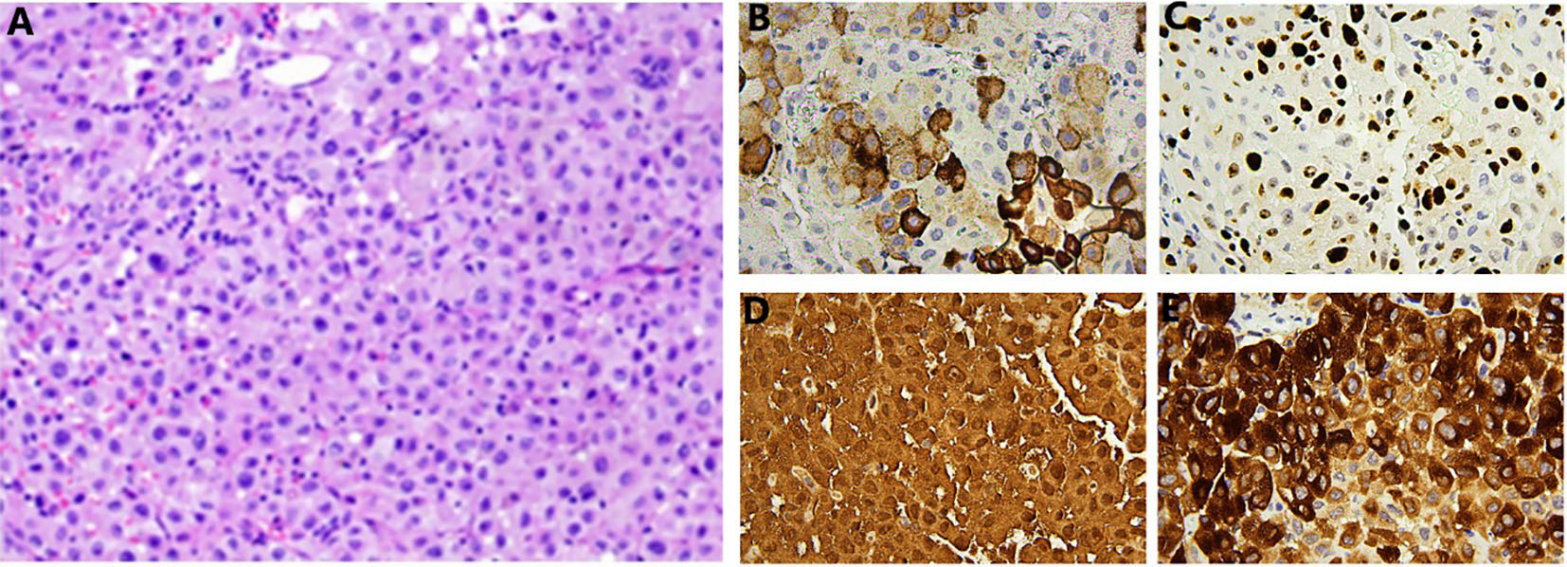
**(A)** HE shows malignant epithelial cells, with diffuse proliferation and infiltration of tumor cells, which are relatively large, HE, ×400. IHC: Biopsy pathology: The cytoplasm is abundant, and the cytoplasm contains abundant melanin; additional spindle-shaped cells can be seen, with slightly irregular shapes and prominent nucleoli. Immunohistochemical staining examination shows that some tumor cells contain a large amount of brown melanin pigment. The lesion cells show **(B)** HMB45(+), EnVision, ×400, **(C)**: Ki-67 (about 30%), EnVision, ×400; **(D)**: S100(+), EnVision, ×400; **(E)**: Melan A (+).

A 59-year-old Asian male was admitted to the hospital due to hematuria. He also presented with frequent urination, urgency, and pain during urination, but had no difficulty in urination. A comprehensive urological ultrasound examination revealed a bladder mass (**Figure 1**), and further urological CT and enhanced CT scans indicated multiple small nodular shadows on both sides of the bladder (**Figure 2**). The patient had a 20-year history of smoking, with 2 packs per day. There were no other systemic diseases, and no medications were taken. Due to gallbladder stones, a cholecystectomy was performed 4 years ago, and the postoperative recovery was good. After the patient was admitted, physical examination showed normal skin color all over the body, with no lesions or discoloration observed. To rule out the possibility of metastasis to the bladder from other primary melanoma lesions, we further conducted CT scans of the entire abdomen, pelvis, and chest to assess for metastatic disease, and no evidence of metastasis was found. After completing the relevant preoperative preparations, a complete transurethral resection of bladder tumor was performed. Further immunohistochemistry was required. To prevent recurrence of bladder tumor, intravesical chemotherapy with epirubicin hydrochloride was subsequently administered (**Table 1**). The immunohistochemistry: A2#: Desmin (-), GATA3 (-), p40 (-), Ki-67 (hotspot area 30%+), p53 (wild type), p63 (-), CK5&6 (-), CK20 (-), CK19 (-), CK7 (-), CK(Pan) (-). (2) Additional immunohistochemistry: A2#: NSE (-), CD56 (patchy +), PSA (-), Vimentin (+), Melan A (diffuse +), HMB45 (patchy +), Synaptophysin (patchy +), CgA (-), S100 (diffuse +), CD31 (-), CD117/c-Kit (-). (**Figure 3**). The final pathology was primary bladder malignant melanoma.

**Table 1:** A structured timeline of diagnosis and treatment.

Time	The process of diagnosis and treatment	Outcomes
2025-10-12	Tentative diagnosis	Bladder cancer
2025-10-15	Surgery interventions	Partial cystectomy
2025-10-23	Confirmed diagnosis	Primary bladder melanoma
2025-10-24	Chemotherapy	Epirubicin hydrochloride
2026-04-20	Follow-up (6Months)	Alive

Follow-up:

The patient was followed up; no recurrence or metastasis was observed.

## Discussion

Malignant melanoma originates from the malignant transformation of melanocytes in the body. These tumors are highly malignant and invasive. The common site of the lesion is the skin, and rarely involves the urinary tract. Wheelock et al. (7) since the first case of primary bladder malignant melanoma was reported in 1942, sporadic cases of this disease have been reported in recent years, but the total number didn’t 50 cases. This cased highlights the significant challenges in diagnosing urological melanoma when typical skin lesions were absent. According to relevant reports, the onset age of this disease was mostly after the age of 50, Male patients had a slightly higher incidence than female patients. This result may be related to the risk factor of men smoking (6). At present, the diagnosis of primary bladder malignant melanoma mainly relies on clinical symptoms and related auxiliary examinations. Physical examination often showed no positive signs. The main symptoms of most patients were hematuria, which was consistent with the clinical manifestations of bladder urothelial tumors (8). Most primary malignant melanoma of the bladder have a black appearance under cystoscopy (9). This tumor has a characteristic brownish or black appearance due to the presence of melanin, but the color may not be uniform; some areas may appear light yellow or gray. However, in the case we reported, no color change was observed in the tumor biopsy. It was consistent with the color of bladder urothelial tumors. This made the diagnosis process more difficult for us. Eventually, through the immunohistochemical results, we concluded: Ki-67 (30%+), CD56 (+), PSA (-), Vimentin (+), Melan A (+), HMB45 (+), Synaptophysin (+), CgA (-), S100 (+), CD31 (-), CD117/c-Kit (-). This was consistent with the previous literature reports (10).

In terms of treatment, we hadn’t a unified treatment plan for primary malignant melanoma. The main reason for this might be that the incidence of this disease is too low, there were few clinical cases, and the prognosis was poor with a relatively low 5-year survival rate (11, 12). The current treatment planed mainly relies on surgical methods, supplemented by postoperative bladder instillation. The surgeries mainly included transurethral bladder tumor resection, partial bladder resection, and radical bladder resection. There are controversies with the treatment. Some studies supported performing radical bladder resection at an early stage, which may increased the patient’s survival time; however, other studies reported that performing transurethral bladder tumor resection at an early stage, followed by postoperative drug instillation therapy, didn’t significantly differ in survival rate from radical resection, and this surgical method can also improve the patient’s quality of life. In our reported case, considering the patient’s enhanced CT results, no metastasis was found in other areas. Therefore, we chosed transurethral bladder tumor resection, followed by bladder instillation with epirubicin hydrochloride. Previous literature reported, gemcitabine (13) and BCG (bacillus Calmette-Guérin) (14) were used for bladder perfusion. Although we didn’t use gemcitabine, after perfusing with epirubicin hydrochloride, the patient hadn’t shown any recurrence during the current follow-up period.

Given the high malignancy of primary malignant melanoma of the bladder, studies have reported that the use of immune checkpoint inhibitors has certain efficacy for this disease. Nivolumab combined with ipilimumab has an effect in blocking PD1, and immunotherapy and targeted therapy can be considered for patients with advanced bladder melanoma (15–17).

There are certain difficulties in the differential diagnosis between primary melanoma of the bladder and bladder urothelial carcinoma. Immunohistochemistry plays a potential role in the differentiation of these two diseases. More clinical data are needed for further research. By reporting this rare case, we aim to provide potential assistance in subsequent treatments.

## Conclusion

Through this case report, we should be more vigilant in our clinical work. Although previous literature indicated that primary malignant melanoma of the bladder usually presents as a visible pigmented mass under cystoscopy, which is relatively easy to identify and pay attention to, the cystoscopic manifestation of this patient was not significantly different from that of common urothelial tumors. It lacks typical features, which brought great difficulties to the diagnosis.

According to related research reports, the diagnosis of primary bladder melanoma mainly relies on immunohistochemical results. Although immunohistochemistry is currently the gold standard for the diagnosis of this disease, future studies may further integrate new diagnostic tools such as gene sequencing technology or analysis of tumor-derived exosomes, with the aim of achieving early detection of this malignant tumor.

Therefore, due to the small number of reported cases, the clinical pathological characteristics, treatment and prognosis of this disease still need further study.

## Data Availability

The original contributions presented in the study are included in the article/supplementary material. Further inquiries can be directed to the corresponding author.

## References

[1] Kozovska Z Gabrisova V Kucerova L . Malignant melanoma: diagnosis, treatment and cancer stem cells. Neoplasma. (2016) 63:510–7. doi: 10.4149/neo_2016_403. PMID: 27268913

[2] Ionescu S Nicolescu AC Madge OL Simion L Marincas M Ceausu M . Intra-abdominal Malignant melanoma: challenging aspects of epidemiology, clinical and paraclinical diagnosis and optimal treatment—a literature review. Diagnostics. (2022) 12:2054. doi: 10.3390/diagnostics12092054. PMID: 36140455 PMC9498106

[3] Hussein MR . Extracutaneous Malignant melanomas. Cancer Invest. (2008) 26:516–34. doi: 10.1080/07357900701781762. PMID: 18568775

[4] Cazzato G Colagrande A Cimmino A Caporusso C Trabucco SMR Zingarelli M . Urological melanoma: a comprehensive review of a rare subclass of mucosal melanoma with emphasis on differential diagnosis and therapeutic approaches. Cancers. (2021) 13:4424. doi: 10.3390/cancers13174424. PMID: 34503234 PMC8431506

[5] Bejrananda T Sawasdee A Boonchai S Tanthanuch M . Primary Malignant melanoma of the bladder: a rare case report in asia and review of the literature. Res Rep Urol. (2021), 833–9. doi: 10.2147/rru.s345322. PMID: 34934756 PMC8684417

[6] Wheelock MC . Sarcoma of the urinary bladder. J Urol. (1942) 48:628–34. doi: 10.1016/s0022-5347(17)70753-7

[7] Dai JD He B Liu ZH Shen PF Shi M . Primary melanoma of the bladder: case report and review of the literature. World J Surg Oncol. (2022) 20:287. doi: 10.21203/rs.3.rs-1479824/v1. PMID: 36071438 PMC9454232

[8] Lamichhane N Dhakal HP . Melanoma of urinary bladder presented as acute urine retention. Nepalese J Cancer. (2017) 1:67–70. doi: 10.3126/njc.v1i1.25637. PMID: 37609976

[9] Safadi A Schwalb S Ben-Shachar I Katz R . Primary Malignant urethral melanoma resembling a urethral caruncle. Urol Case Rep. (2017) 15:28–9. doi: 10.1016/j.eucr.2017.08.004. PMID: 28932693 PMC5596331

[10] Karabulut YY Erdogan S Sayar H Ergen A Baydar DE . Primary Malignant melanoma of the urinary bladder: clinical, morphological, and molecular analysis of five cases. Melanoma Res. (2016) 26:616–24. doi: 10.1097/cmr.0000000000000300. PMID: 27603550

[11] Díaz-Fuentes H Fuziwara-Ruíz S de Los Santos-Frias E Amaya-Fragoso E Rodríguez-Araujo E Ramos-Arceo E . Primary Malignant melanoma of the bladder: a rare case report. Urol Case Rep. (2025), 103240. doi: 10.1016/j.eucr.2025.103240. PMID: 41142186 PMC12549713

[12] Barillaro F Camilli M Dessanti P Gorji N Chiesa F Villa A . Primary melanoma of the bladder: case report and review of the literature. Arch Ital Urol Andrology/Archivio Italiano di Urol Andrologia. (2018) 90(3). doi: 10.4081/aiua.2018.3.224. PMID: 30362694

[13] Su ZT Florissi IS Geller AE Johnson BA III Hoffman-Censits JH Patel SH . Cost-effectiveness of perioperative durvalumab with neoadjuvant gemcitabine/cisplatin in muscle-invasive bladder cancer treatment. J Natl Compr Cancer Network. (2026) 1:1–7. doi: 10.6004/jnccn.2025.7129. PMID: 41911914

[14] Rapisarda S Bada M Polara A Crocetto F Creta M Chiancone F . Conservative management of primary Malignant melanoma of the bladder: a case report. J Med Case Rep. (2021) 15:39. doi: 10.1186/s13256-020-02602-7. PMID: 33541425 PMC7863235

[15] Venyo AKG . Melanoma of the urinary bladder: a review of the literature. Surg Res Pract. (2014) 2014:605802. doi: 10.1155/2014/605802. PMID: 25374957 PMC4208590

[16] Pfail JL Katims AB Alerasool P Sfakianos JP . Immunotherapy in non-muscle-invasive bladder cancer: current status and future directions. World J Urol. (2021) 39:1319–29. doi: 10.1007/s00345-020-03474-8. PMID: 33057888

[17] Schindler K Schicher N Kunstfeld R Pehamberger H Toepker M Haitel A . A rare case of primary rhabdoid melanoma of the urinary bladder treated with ipilimumab, an anti-CTLA 4 monoclonal antibody. Melanoma Res. (2012) 22:320–5. doi: 10.1097/cmr.0b013e32835566c0. PMID: 22713795

